# The use of a thin guide‐wire for urethral definition in prostate SBRT treatments with Cyberknife

**DOI:** 10.1002/acm2.14006

**Published:** 2023-04-25

**Authors:** David Sevillano, Asunción Hervás, Juan D. García‐Fuentes, Carmen Vallejo, Fernando López, Rafael Colmenares, Ana Belén Capuz, Rafael Morís, Miguel Cámara, Pablo Galiano, Sandra Williamson, Rubén Chillida, María Josefa Béjar, Daniel Prieto, Feliciano García‐Vicente

**Affiliations:** ^1^ Medical Physics Department Hospital Universitario Ramón y Cajal, IRYCIS Madrid Spain; ^2^ Radiation Oncology Department Hospital Universitario Ramón y Cajal, IRYCIS Madrid Spain

**Keywords:** Cyberknife, prostate SBRT, urethral contouring

## Abstract

**Purpose:**

To study and analyze the effect of the use of a thin guide‐wire instead of a Foley catheter for urethral definition in prostate stereotactic body radiation therapy (SBRT) treatments and to compare treatment parameters in both situations.

**Material and Methods:**

Thirty‐seven prostate SBRT patients were employed in this study. A Foley catheter was employed in nine of them, and a guide‐wire was employed in the other 28 patients. For each of the 28 patients in which the guide‐wire was employed, a comparison between urethral positions in both situations was performed, allowing for a margin definition of the urethra when a Foley catheter was employed. Displacements of the prostate during treatment were obtained, allowing for an analysis of prostate positions in both situations. Also, different treatment parameters such as the number of treatment interruptions, couch movements performed, and x‐rays needed were gathered.

**Results:**

Large differences between urethral positions can be found in the anterior‐posterior (AP) directions compared to those in the lateral (LAT) direction. Differences are also larger in areas closer to the base of the prostate, where margins applied in the case of using a Foley catheter are 16 mm with a mean displacement of 6 mm in the posterior direction. No differences in the treatment parameters were found during treatment in both situations. The difference found in absolute prostate pitch rotations suggests that the Foley catheter provokes a shift of the prostate position, which does not occur when employing the guide‐wire.

**Conclusions:**

Foley catheters shift the urethral position, making them a wrong surrogate of the urethra when no catheters are present. The margins needed to assess uncertainties introduced by the use of a Foley catheter are larger than those usually applied. The use of a Foley catheter did not present any additional difficulty during treatment delivery in terms of images employed or interruptions produced.

## 1 INTRODUCTION

Many prostate radiotherapy protocols imply the delivery of high biological equivalent doses that may involve the appearance of genitourinary (GU) complications. It is thus recommended to contour the urethra in procedures such as prostate brachytherapy,^1^ stereotactic body radiation therapy (SBRT) of the prostate,^2^ or focal boost treatments in the prostate.^3^ Registration of the dose received by the urethra in such procedures helps to spare this organ and reduce possible GU toxicities.^4^, ^5^

The most common method to contour the urethra with the standard equipment present in a radiation oncology department is the use of a Foley catheter that allows urethral visualization in a conventional computed tomography (CT) scan. This method, however, presents many problems related to the perturbations such catheters provoke in urethral and prostatic positions,^6^, ^7^ which are more important if the catheter is not used during treatment delivery. Magnetic resonance (MR) can also be employed for urethra contouring,^8^, ^9^, ^10^ although the use of MR might not be possible as a standard procedure for prostate treatments and the presence of fiducial markers might aggravate visualization of the urethra.

The use of a thin guide‐wire instead of a catheter has been proposed in previous works.^7^, ^11^ The work by Shimizu et al. did not perform a deep study of the geometrical implications of the use of Foley catheters. Dekura et al. studied the variations of urethral positions in both situations by gathering data from three different sectors of the prostate.

In our work, data of the differences in the position of the urethra depending on the surrogate used to contour it are obtained and a method for creating a planning organ at risk volume (PRV) is proposed. Additionally, rotations of the prostate during treatment and several treatment parameters of treatments performed using both methods are shown.

## 2 MATERIALS AND METHODS

### 2.1 Patients

Thirty‐seven patients were analyzed in this work. Nine of them were simulated with a Foley catheter to delineate the urethra before the thin guide‐wire procedure was implemented. The other 28 patients had two CT scans, one with the Foley catheter and one with the thin guide‐wire, which was employed for planning. In both situations, the patients were treated without a catheter or a guide‐wire.

### 2.2 Catheter placement

Patients imaged with the Foley catheter underwent a unique CT scan used for planning.

In those patients in which a guide‐wire was employed, the procedure was as follows:

Firstly, a Foley catheter with the guide‐wire inside was inserted into the urethra, and a CT scan was performed. Once it was observed that the catheter was correctly placed, the Foley catheter was removed from the patient, leaving the guide‐wire in the urethra. Then, a second CT scan was performed and used for planning.

**FIGURE 1:**
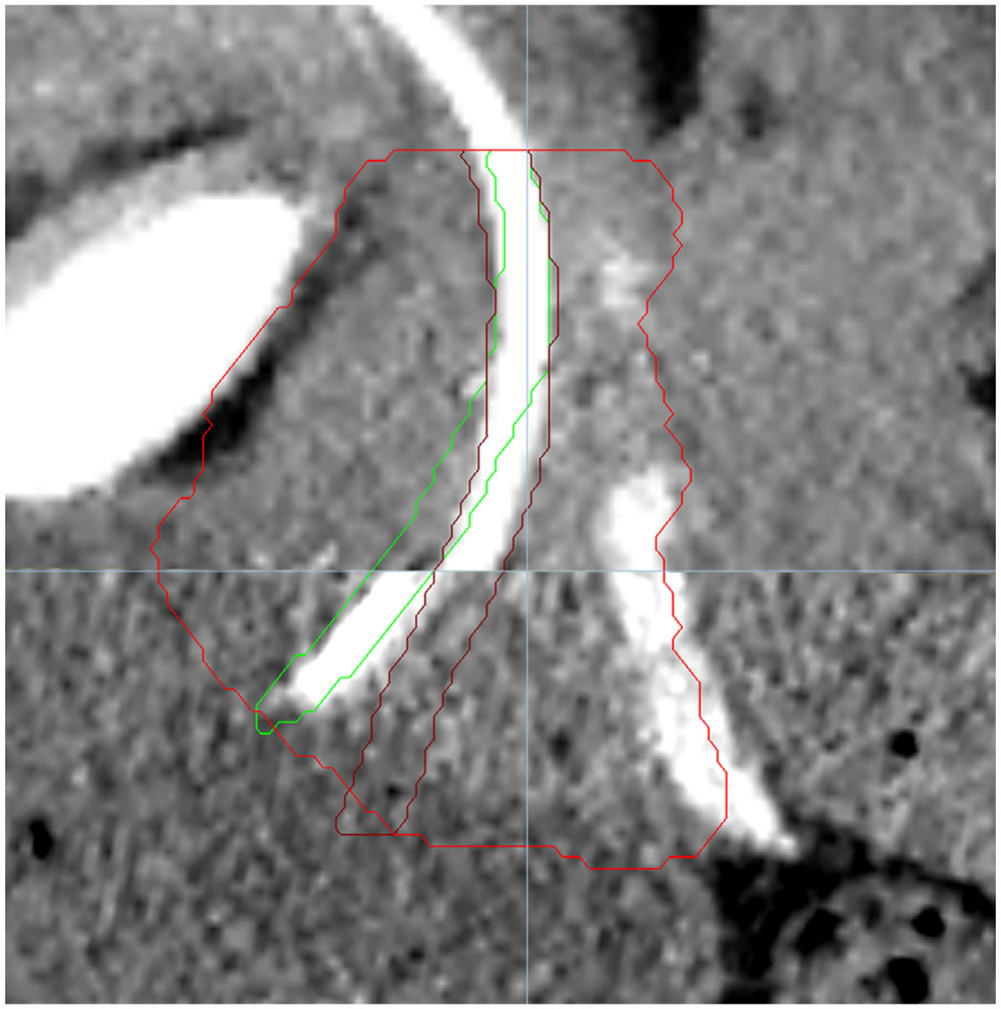
Example of the displacement of the urethra when a thin guide‐wire (upper part of the picture) and a Foley catheter (lower part of the picture) are employed.

Both scans were imported to Precision software (Accuray, USA) and registered based on the four fiducial markers placed in the prostate correcting for both translations and rotations. The urethra was then contoured on each scan. An example of the registration of both CT scans and urethral displacement is shown in Figure 1.

### 2.3 Treatment planning and delivery

Two coupled gold fiducial markers with dimensions of 3 mm × 1 mm and a separation of 20 mm (QLRAD, FL, USA) were inserted in the prostate gland 1 week before the planning CT scan. 36.25 Gy was prescribed in five fractions to the PTV defined as the prostate gland and the proximal seminal vesicles plus a 5 mm margin. The urethra was contoured at each slice as an 8 mm‐diameter circumference with its center at the surrogate employed (Foley catheter or guide‐wire). Only those slices containing the PTV were employed. Treatment was planned and delivered in a Cyberknife (Accuray, CA, USA) system with a multileaf collimator.

The Cyberknife system allows intrafraction control of translations and rotations of the prostate to be performed and automatically corrects for them provided that the prostate position is within a certain tolerance. In the case of prostate treatments, the system is capable of correcting rotations within ± 5⁰ in pitch and ± 3⁰ in roll and yaw. Therefore, it is important to set the patient up within these limits to allow for rotation corrections during treatments.

### 2.4 Internal motion analysis

For those patients with a thin guide‐wire, contours of the urethra with and without the Foley catheter were exported to an in‐house software in which the analysis was performed. Only the contours in which the urethra was inside the PTV were employed.

Centroid positions of the urethra with the Foley catheter at each slice were compared to those with the guide‐wire, which was used as a reference. Thus, values of their differences for each longitudinal position were available. Those longitudinal positions were transformed in 20 positions from 0 to 100, representing the apex and the base of the prostate, respectively. This way, differences between urethral positions of different patients could be determined.

The distribution of differences in the anterior‐posterior (AP) and lateral (LAT) directions at each longitudinal position for each patient was obtained. For each prostate segment, mean displacement and standard deviation were calculated.

### 2.5 Planning organ at risk volume (PRV) margin

By obtaining the distribution of differences between positions of the urethra at each prostate sector with and without a Foley catheter, it is possible to propose the PRV margins that should be employed when a Foley catheter is used for urethral definition. Thus, the PRV of the urethra would be a set of ellipses of different sizes at each prostate sector with its center displaced according to the mean displacements found in the population. To assure that the PRV covers 90% of the population, the axis of the ellipses will be the standard deviation of the differences found multiplied by a factor of 2.15.

### 2.6 Treatment performance

Log files of each treatment fraction were extracted to allow the analysis of the effect of the use of both surrogates on the treatment performance. Data of intratreatment prostate rotations and couch rotations were obtained. Also, the number of images taken before treatment, the total number of images, the number of couch shifts, and the number of interruptions during treatment were gathered.

Absolute prostate rotations were calculated as the sum of rotations applied to the couch and prostate rotations assessed by the tracking system. These two parameters allow for the assessment of whether there is any systematic error produced by catheter placements on the average prostate position. For each patient group (Foley catheter and guide‐wire), mean error (μ), systematic error (Σ), and random errors (σ) of couch and prostate rotations were obtained. Also, a global analysis was performed from all the images obtained from each patient group. Differences between groups were analyzed with a one‐tail Student´s *t*‐test.

A number of images, treatment interruptions, and couch shifts were expressed per treatment session for each group. The number of treatment sessions was analyzed per patient.

It should be noted that in this analysis, an interruption occurs if there is any delivery interruption or if the patient has to leave the treatment room as long as the treatment session is completed that same day. A session occurs if a treatment fraction could not be completed or even started 1 day and the patient had to come another day to be treated. This happens if it is not possible to align the prostate position to allow for rotation corrections during treatment due to variations in the internal anatomy of the patient.

## 3 RESULTS

### 3.1 Internal motion analysis

**FIGURE 2:**
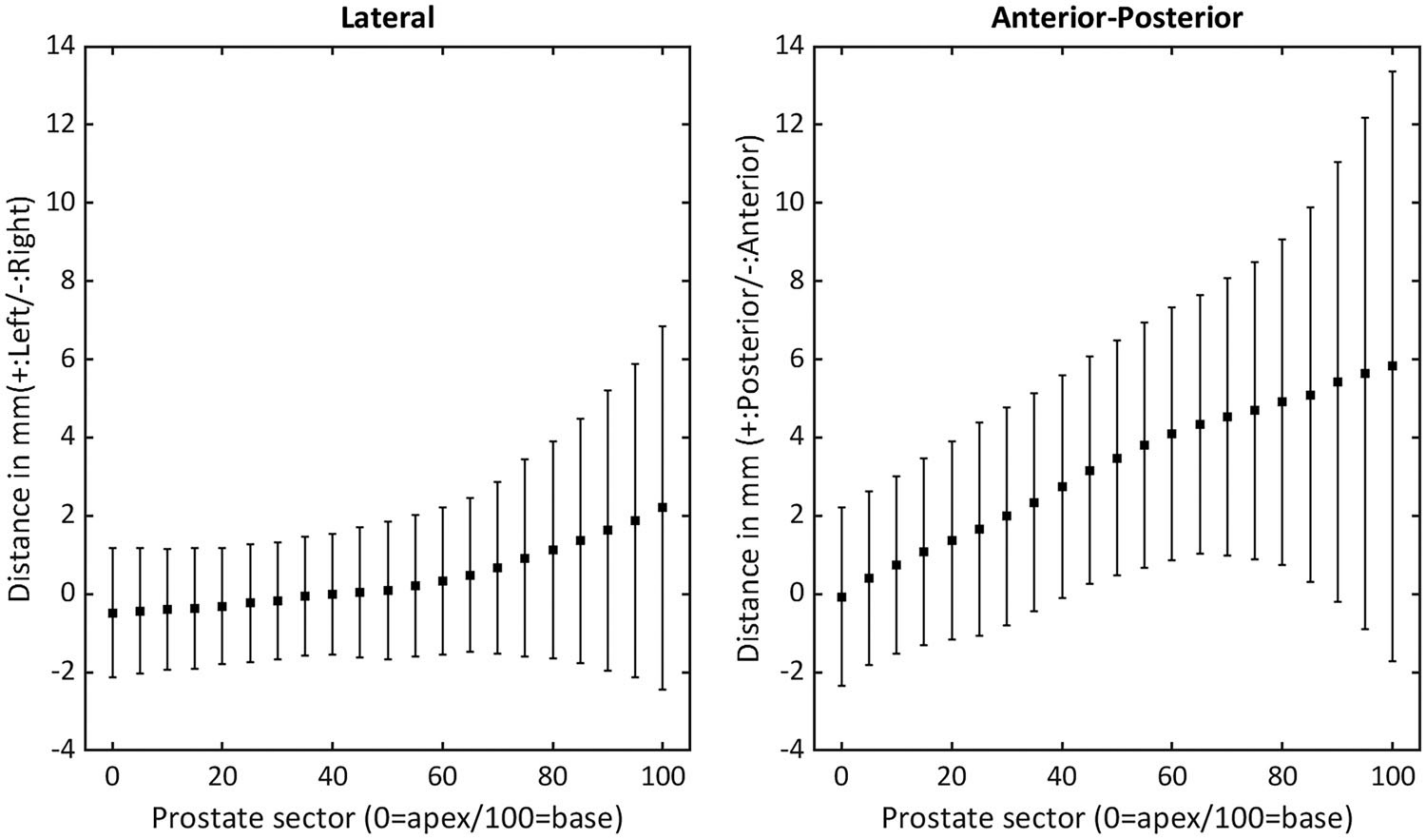
Mean values of the differences in urethral position when a Foley catheter and a thin guide‐wire are employed at each prostate sector. Error bars represent 1 SD.

**FIGURE 3:**
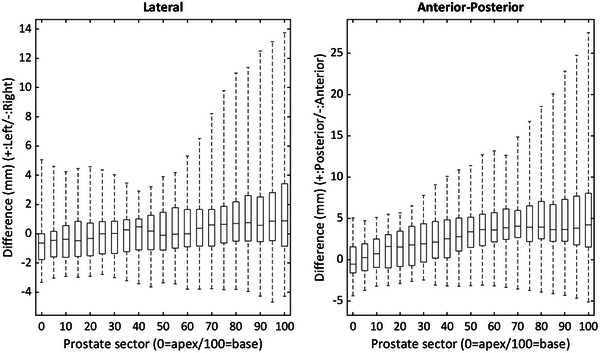
Box plot of the differences between urethral positions at each prostate sector. The plot shows median value, limits of 25% and 75% quartiles, and range of each value.

Mean differences and standard deviations between urethra positions with and without a Foley catheter per prostate sector are shown in Figure 2. Also, for ease of comparison to the work of Dekura et al.,^7^ a boxplot of differences at each sector is shown in Figure 3. These differences increase in magnitude as the urethra gets closer to the bladder, being more important in the AP direction than in the LAT direction. The variability of these differences is also increased in the most cranial sectors.

These data are also shown in Table 1, where margins for a urethral PRV in the case that a Foley catheter is used for delineation of the urethra are included. These margins are designed to cover 90% of the patient population. As mentioned above, the final PRV at each prostate sector would be comprised of ellipses with their axes equal to the margins calculated in Table 1 and their centers displaced according to the mean differences of both urethral contours at each sector.

**TABLE 1:** Mean differences, standard deviation, and margins needed to apply in the case that a Foley catheter is used to represent the urethra.

Prostate sector		0	10	20	30	40	50	60	70	80	90	100
Mean difference (mm)	LAT	−0.5	−0.4	−0.3	−0.2	0.0	0.1	0.3	0.7	1.1	1.6	2.2
	AP	−0.1	0.7	1.4	2.0	2.7	3.5	4.1	4.5	4.9	5.4	5.8
Standard deviation (mm)	LAT	1.6	1.5	1.5	1.5	1.5	1.8	1.9	2.2	2.8	3.6	4.6
	AP	2.3	2.3	2.5	2.8	2.8	3.0	3.2	3.6	4.2	5.6	7.5
Margin (mm) (2.15σ)	LAT	3.5	3.3	3.2	3.2	3.3	3.8	4.0	4.7	6.0	7.7	10.0
	AP	4.9	4.8	5.4	6.0	6.1	6.4	7.0	7.6	8.9	12.1	16.2

Abbreviations: AP, anterior‐posterior; LAT, lateral.

### 3.2 Treatment performance

Data population of prostate and couch rotations for both groups are shown in Table 2. Differences between both patient groups were not significant for roll and yaw rotations, while for pitch, a significant difference was found in the systematic displacement for both couch (*p* = 0.05) and absolute prostate (*p* = 0.03) rotations. As treatments are performed in both cases without catheters, it is expected that both systematic (Σ) and random errors (σ) be equal.

**TABLE 2:** Population data of couch and absolute prostate rotations in treatments with the Foley catheter and thin guide‐wire.

	Foley catheter (*n* = 9)					Guide‐wire (*n* = 28)				
	Couch		Prostate			Couch		Prostate		
	Roll	Pitch	Roll	Pitch	Yaw	Roll	Pitch	Roll	Pitch	Yaw
μ (mm)	−0.12	0.94	−0.03	1.7	0.17	0.22	−0.08	0.84	−0.21	0.4
Σ (mm)	1.0	1.70	1.3	2.8	0.86	0.77	1.2	0.6	2.1	0.5
σ_inter_ (mm)	0.25	0.39	0.18	1.28	0.27	0.41	0.59	0.51	1.34	0.48
σ_intra_ (mm)	0.53	0.66	0.7	1.5	0.65	0.5	0.6	1.6	2.1	1.4

The results for the rest of the treatment parameters (mean pretreatment images, mean total number of images, mean number of couch shifts, and mean number of sessions) are shown in Table 3. All of the parameters except the pretreatment images did not show significant differences between both patient groups. For the pretreatment images, it was found that for patients with the guide‐wire, more images were needed to obtain a correct setup before treatment.

**TABLE 3:** Treatment parameters for both patient populations. The *p*‐value in parenthesis represents the significance of the differences between the standard deviations of both populations.

	Mean value (SD)		
	Foley catheter	Guide‐wire	*p*‐value
Pretreatment images	6.3 (2.1)	8.1 (4.3)	0.05 (0.02)
Total images	45.2 (10.9)	49.1 (11.6)	0.2 (0.5)
Interruptions	6.3 (6.0)	6.8 (6.6)	0.4 (0.4)
Couch shifts	7.0 (5.3)	7.2 (3.5)	0.5 (0.05)
Sessions	5.9 (0.8)	5.7 (1.2)	0.3 (0.1)

**FIGURE 4:**
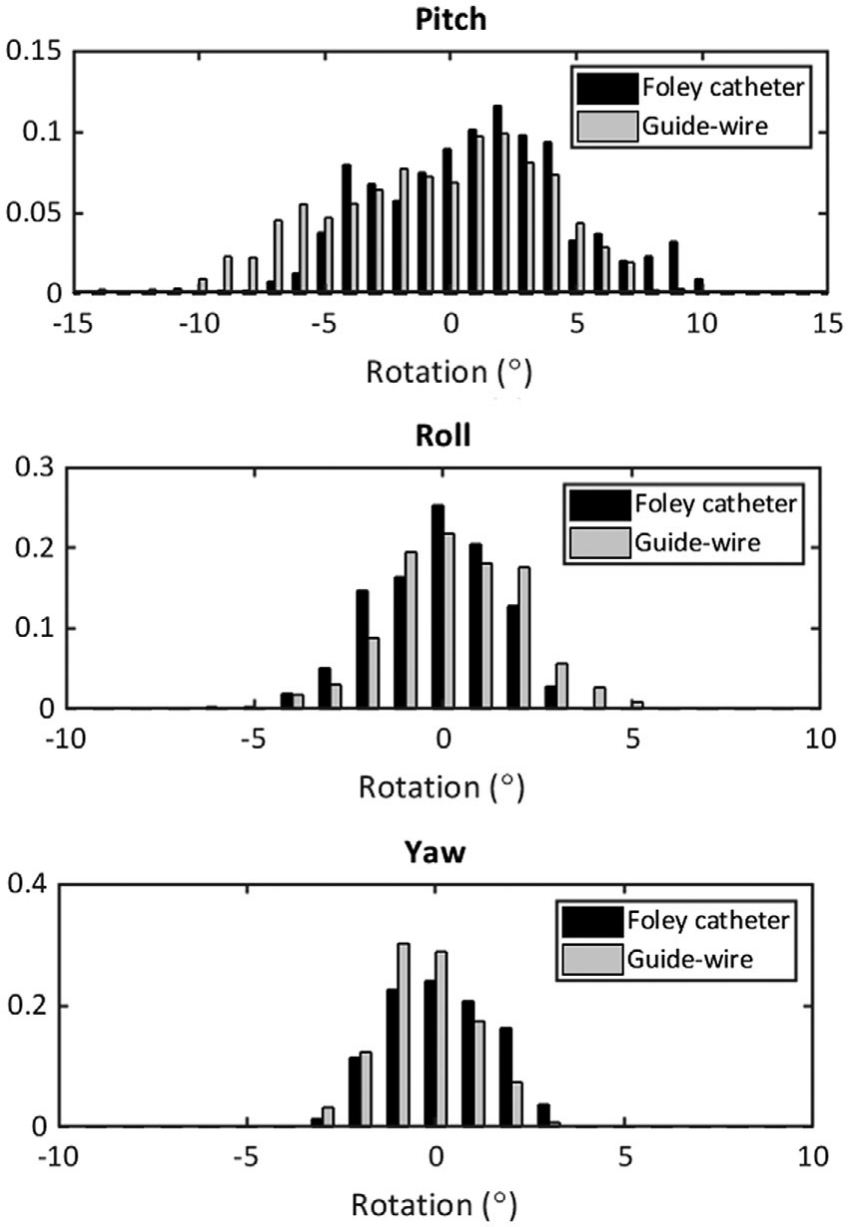
Histogram of prostate rotations when using a Foley catheter (black) and a thin guide‐wire (grey) as a surrogate of the prostate.

The histograms of prostate rotations in both patient populations are shown in Figure 4. In this case, the analysis is performed for all images taken during treatment. Again, it is shown that the distributions of the roll and yaw rotations are almost equal, although a small shift between the distributions is found for the pitch rotation. This is compatible with the data shown in the patient population analysis.

## 4 DISCUSSION

The results on the use of a thin guide‐wire compared to a Foley catheter as a surrogate of the urethra in prostate SBRT are presented in this work. Previously, differences between urethra position with a Foley catheter and a guide‐wire were published by Dekura et al.^7^ In that work, the prostate was divided in three sectors (superior, middle, and inferior segments), and while medians of the differences were not important (1.4 mm in the AP direction in the middle and superior segments), the range of these differences was very large (15 mm in the AP direction and 10 mm in the LAT direction). In that study, the data were presented in a way that was not easily applicable to a definition of a PRV. In our work, with much less patients, larger systematic differences are observed in our population in those superior sectors of the prostate (up to 5 mm in the AP direction and 2 mm in the LAT direction). Also, the range of differences is much larger (−4.7 mm and 13.7 mm in the LAT direction and −5.1 mm and 27.5 mm in the AP direction).

This work presents a method to define different PRV margins of the urethra at each prostate sector, which is a capability not present in commercial treatment planning systems (TPSs). However, it could be possible to create the structures with an external software prior to treatment planning.

One of the expected results in patients simulated with a thin guide‐wire was the improvement of the treatment parameters and easiness of the treatment delivery, as the guide‐wire would have less impact on the prostate position compared to a Foley catheter.^6^ We have detected that in patients in which the urethra was imaged with a guide‐wire, mean value of pitch rotations during treatment was smaller compared to those with a Foley catheter. This difference is significant at the 95% confidence level, and this significant difference is also observed in the couch rotations applied during treatment. Nevertheless, no other parameters show an improvement in the treatment performance of patients with a thin guide‐wire. In fact, the number of images taken prior to treatment in order to set up the patient within the rotation range that allows rotation corrections is higher in those patients simulated with a thin guide‐wire. It should be noted that many factors might affect prostate position prior to treatment, such as adequate bladder and rectal filling or therapist experience, which may explain this result.

However, these data (the absence of a systematic pitch rotation) suggest that the thin guide‐wire is a better surrogate of the urethra than the Foley catheter, as it would provoke fewer variations in the prostate rotation. That might imply that the thin guide‐wire represents a more realistic position of the urethra within the prostate than Foley catheters.

## 5 CONCLUSIONS

Foley catheters shift the urethral position, making them a wrong surrogate of the urethra when no catheters are present. The data of prostate rotations during treatment show that the catheter also produces a systematic shift in the prostate position and that this is not the case when a thin guide‐wire is employed, suggesting that the latter is a better surrogate of the urethra. The use of a Foley catheter did not present any additional difficulty during treatment delivery in terms of images employed or interruptions produced.

## AUTHOR CONTRIBUTIONS

Conceptual design of the study was carried out by David Sevillano and Asunción Hervás. Writing of the manuscript, analysis, and software development was performed by David Sevillano. Asunción Hervás, Fernando López, and Carmen Vallejo performed contouring tasks. Juan D. García‐Fuentes, Rafael Colmenares, Ana Belén Capuz, Rafael Morís, Miguel Cámara, Pablo Galiano, Sandra Williamson, Rubén Chillida, María Josefa Béjar, and Daniel Prieto contributed to data gathering and analysis. Feliciano García‐Vicente performed critical revision of the manuscript.

## CONFLICT OF INTEREST STATEMENT

The authors declare no conflicts of interest.
